# Pd(II)/HPMoV-Catalyzed Direct Oxidative Coupling Reaction of Benzenes with Olefins

**DOI:** 10.3390/molecules15031487

**Published:** 2010-03-09

**Authors:** Yasushi Obora, Yasutaka Ishii

**Affiliations:** Department of Chemistry and Materials Engineering, Faculty of Chemistry, Materials and Bioengineering and High Technology Research Center, Kansai University, Suita, Osaka 564-8680, Japan

**Keywords:** direct coupling, oxidative coupling, arenes, molybdovanadophosphoric acid

## Abstract

The direct aerobic coupling reaction of arenes with olefins was successfully achieved by the use of Pd(OAc)_2_/molybdovanadophosphoric acid (HPMoV) as a key catalyst under 1 atm of dioxygen. This catalytic system could be extended to the coupling reaction of various substituted benzenes with olefins such as acrylates, aclrolein, and ethylene through the direct aromatic C-H bond activation.

## 1. Introduction

The transition-metal-catalyzed arylation of olefins with aryl halides or triflates, which referred to as the Mizoroki-Heck reaction, is an important and a highly versatile C-C bond transformation for the synthesis of arene derivatives [1,2]. However, this existing methodology does not avoid the formation of undesired waste salts arising from the use of aryl halides and bases. Therefore, the development of the direct oxidative couplings of arenes with olefins to afford alkenyl arenes without formation of any salts is highly desired, especially from the environmental and industrial point of view [3,4,5,6,7].

The first waste-free oxidative coupling of arenes was achieved by Fujiwara and Moritani by the stoichiometric reaction of benzene with styrene-palladium chloride dimer in AcOH to give stilbenes in 24% yield [8]. Thereafter, the same group presented the catalytic aerobic oxidative Mizoroki-Heck type reaction by using Pd(OAc)_2_ combined with Cu(OAc)_2_ under elevated oxygen pressure (50 atm) [9]. After several modified versions of this prototype reactions were reported [10,11,12], several practical advances in the development of the catalytic method was evident in recent years [13,14,15]. For instance, Kitamura and Fujiwara reported that the use of a stoichiometric amount of *tert*-butyl hydroperoxide as the oxidant in the Pd(OAc)_2_ combined with benzoquinone as catalysts led to a significant development in more active reoxidation system [13]. Furthermore, Jacobs and coworkers found that the addition of small amounts (4–20 mol %) of Mn(OAc)_3_ or benzoic acid are effective for the Pd(II)-catalyzed oxidative Mizoroki-Heck type reaction under 8 atm of O_2_ [14]. This methodology has been recently extended to the Pd (II)-catalyzed C-H bond alkenylation of anilides and heteroarenes such as pyrroles and indoles [15,16,17].

In this review, we summarize recent progress from our laboratory in terms of the aerobic oxidative coupling reaction of arenes with olefins such as acrylates [18,19], α,β-unsaturated aldehydes [20,21] as well as ethylene [22] under ambient dioxygen atmosphere by using Pd(OAc)_2_/molybdovanado-phosphoric acid (HPMoV) as a key catalyst system. 

## 2. Results and Discussion

### 2.1. Oxidative Mizoroki-Heck type coupling reaction of arenes with acrylates

The reaction of benzene (**1a**) with ethyl acrylate was chosen as a model reaction and carried out under various reaction conditions (Equation 1). The results are shown in Table 1.

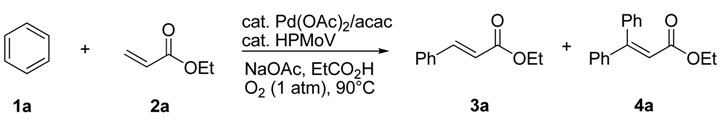
(1)

The coupling reaction of **1a** (30 mmol) and **2a** (1.5 mmol) catalyzed by Pd(OAc)_2_ (0.1 mmol, 6.7 mol %) combined with H_4_PMo_11_VO_40_·30H_2_O (HPMo_11_V_1_, 0.02 mmol, 1.3 mol %) proceeded smoothly in the presence of small amounts of acetylacetone (0.1 mmol, 6.7 mol %) and NaOAc (0.08 mmol, 5.3 mol %) under 1 atm of O_2_ in propionic acid at 90 °C for 2.5 h to give ethyl cinnamate (**3a**) and ethyl β-phenylcinnamate (**4a**) in 74% and 13% yields, respectively. In this reaction, ethyl 3-propionyloxyacrylate (**5**) derived from **2a** and propionic acid was obtained in 4% as by-products (entry 1). The product distribution of **3a** and **4a** was controlled by the reaction time. When the reaction time was prolonged to 5 h, **4a** was formed in good yield (81%) as a major adduct (entry 2). Removing of NaOAc or acetylacetone from the present catalytic system resulted in the decrease of the coupling products, **3a** and **4a** (entries 3 to 4). It is considered that acetylacetone would be functioned as a ligand in the catalyst system, and the addition of acetylacetone is not necessary when Pd(acac)_2_ was employed in place of Pd(OAc)_2_ (entry 5). The use of excess amount of **1a** is favorable and when the amount of **1a** was reduced from 30 mmol to 6 mmol, the yield of **3a** decreased to 51% and the yield of **5** was increased from 4% to 16% (entry 6). As for a choice of a solvent in this reaction, the use of acetic acid instead of propionic acid resulted in considerable decrease of the yields of **3a** and **4a** (entry 7). The yield of coupling products, **3a** and **4a**, was markedly influenced by the combination of Pd(OAc)_2_ with molybdovanadophosphoric acids (HPMoV) which serve as reoxidation catalysts of the reduced Pd(0) to Pd(II) during the reaction course. Thus, the reaction was sluggish in the absence of HPMoV (entry 8). The performance of various heteropoly acid including HPMoV as reoxidation catalysts was affected by the vanadium content in the heteropolyacid (entries 9 to 12). The reaction by the use of 12-molybdophosphoric acid (HPMo_12_) not involving V ion brought about **3a** in low yield (52%) (entry 12). In addition, the effect of several alkali metal acetates as a base, was examined. As a result, NaOAc was found to be the best base in the present catalytic system (entry 1 *vs.* entries 13–16). One of the most important feature of this method is that the reaction smoothly proceeded under 1 atm of O_2_. Moreover, it is useful from the practical synthetic viewpoint that the reaction tolerated under air in place of pure O_2_, and **3a** was obtained in 74% yield for 5 h (entry 17). However, the reaction in the absence of dixoygen, namely under Ar (1 atm), was difficult to take place (entry 18).

Various arenes (**1**) were allowed to react with acrylates **2** under the optimized reaction conditions. These results are summarized in Table 2. Benzene (**1a**) reacted smoothly with several acrylates **2b**-**2e** to give the corresponding cinnamates **3b-3d** in 68–73% yields with concomitant formation of β-phenylcinnamate in 11–16% yields (entries 1 to 3). The arylation of 4-phenyl-2-butenone (**2e**) under these reaction conditions gave 1,1-diphenyl-3-butenone (**3e**) in 93% yield (entry 4). Reaction of various arenes with ethyl acrylate (**2a**) was also carried out under optimized reaction conditions (entries 4-10). The reaction of toluene (**1b**) with **2a** afforded the coupling products **3f** in 70% total yield as a isomeric mixture consisting of *o*-**3f** : *m*-**3f** : *p*-**3f** = 15:41:44 (entry 5). 

Anisole (**1c**) was subjected to react with **2a** at 60 °C to give a mixture of coupling products **3g** in 73% total yield (entry 6). The reaction of chlorobenzene (**1d**) and bromobenzene (**1e**) with **2a** gave the corresponding oxidative coupling products, **3h** and **3i** exclusively, in 74% and 49% yields, respectively. It is noteworthy that the reaction of **1d** and **1e** gave oxidative coupling products exclusively, in preference to the products based on the Mizoroki-Heck reaction (entries 7 and 8). The reaction of 1,2-dimethoxybenzene (**1f**) with **2a** afforded ethyl 3,4-dimethoxycinnamate (**3j**) in 67% yield (entry 9). Unfortunately, the reaction of 2-methyl-thiophene (**1g**) with **2a** under the above-mentioned reaction conditions did not take place at all. Alternatively, Pd(OAc)_2_ supported on active carbon, Pd(OAc)_2_/C, was efficient for the coupling of **1g** with **2a** to give the corresponding coupling product, **3k**, in 86% yield (entry 10). 

In this reaction, reoxidation step is important to determine the turn-over number (TON) of the present reaction, which was markedly affected by the choice of HPMoV catalysts. Therefore, the reaction of **1a** (45 mmol) and **2a** (3 mmol) was carried out in the presence of Pd(OAc)_2_ (0.03 mmol), HPMoV (0.02 mmol), NaOAc (0.08 mmol), acetylacetone (0.03 mmol), and EtCOOH (5 mL) under O_2_ (1 atm) at 90 °C for 12 h. The results of TONs for the formation of **3a** and **4a** by using various HPMoV under these conditions were as follows: 19 for H_3_PMo_12_O_40_·30H_2_O, 76 for H_4_PMo_11_VO_40_·30H_2_O, 10 for H_5_PMo_10_V_2_O_40_·28H_2_O, 8 for H_6_PMo_9_V_3_O_40_·30H_2_O, and 6 for H_7_PMo_8_V_4_O_40_·28H_2_O. 

We previously reported that combined catalysts of Pd(OAc)_2_ with HPMo_11_V_1_ and HPMo_12_ as the reoxidation catalyst was the most active for the Pd-catalyzed oxidative coupling of benzene to biphenyl under O_2_ (1 atm) [23]. In the present reaction, the best TON (121) was observed by the reaction in the presence of Pd(OAc)_2_ combined with a 1:1 mixture of HPMo_11_V_1_ and HPMo_12_ under these reaction conditions. 

To obtain mechanistic information on the present reaction, the initial rate of the reaction of benzene **1a** with **2a** was compared with that of benzene-*d*_6_ (**1a_-*d*_**) with **2a** under the same conditions as entry 1 in Table 1. It was found that a kinetic isotope effect for the coupling reaction was evident and the rate of the reaction of **1a** with **2a** was four times faster than that of benzene-*d_6_* (**1a_-*d*_**) with **2a**, *i*.*e*., *k*_H_/*k*_D_ ≈ 4. The same results (*k*_H_/*k*_D_ ≈ 4) were obtained by the reaction of a 1:1 mixture of **1a** and **1a_-*d*_** with **2a** under these conditions. These facts indicate that the cleavage of the C-H bond of the **1a** is the rate-determining step in a sequence of reactions.

**Scheme 1:**
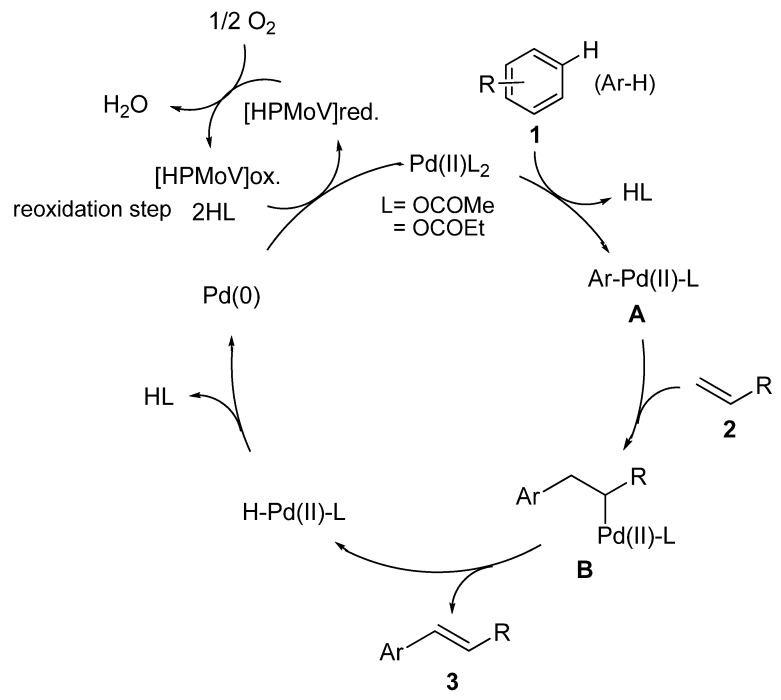
A possible reaction path for the coupling of arene with acrylate.

Although a detailed reaction mechanism remains to be further elucidated, it is possible to explain the reaction rationally by a pathway similar to that proposed by Kitamura and Fujiwara [13]. A plausible reaction path is shown in Scheme 1. First, electrophilic attack of a Pd(II) to arene **1** would initiate the reaction, leading to a σ-aryl-palladium (II) intermediate (**A**), which is considered to be the rate-determining step by judging from the labeled experiment (vide supra). The insertion of olefins like acrylate **2** to σ-aryl-palladium (II) intermediate (**A**) takes place to give σ-alkyl-palladium (II) intermediate **B**. Subsequently, *β* -hydride elimination of the **B** gives the desired coupling products **3** along with Pd-H intermediate. The Pd-H intermediate is reduced to Pd(0), and the resulting Pd(0) species is reoxidized by [HPMoV]ox. to generate initial Pd(II) catalyst. The [HPMoV]red. is easily oxidized to [HPMoV]ox. with atomospheric dioxygen.

### 2.2. Oxidative Mizoroki-Heck type coupling of benzenes with α,β-unsaturated aldehydes

The oxidative coupling reaction of benzene (**1a**) with α,β-unsaturated aldehydes such as acrolein (**6a**) was also achieved by slight modifications of the reaction conditions as reported in section 2.1, which leads to cinnamaldehyde (**7a**) and *β*-phenyl cinnamaldehyde (**8**) as products (Equation 2).

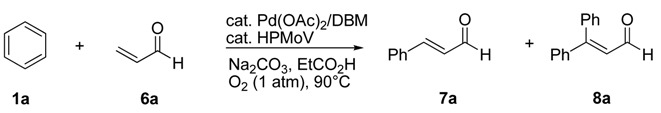
(2)

The results of the reaction of **1a** with **6a** under various conditions are summarized in Table 3. The best result for the formation of cinnamaldehyde (**7a**) was attained by the reaction conditions as shown in Table 3, entry 1. Namely, a mixture of **1** (30 mmol) and **6a** (1.5 mmol) was allowed to react under the influence of Pd(OAc)_2_ (0.1 mmol, 6.7 mol %), H_4_PMo_11_VO_40_·26H_2_O (HPMo_11_V_1_, 0.02 mmol, 1.3 mol %), Na_2_CO_3_ (0.05 mmol, 3.4 mol %), and dibenzoylmethane (DBM) (0.1 mmol, 6.7 mol %) under O_2_ (1 atm) in propionic acid (5 mL) at 90 °C for 1.5 h, giving **7a** in 59% yield along with **8** (5%). In the present reaction, a trace amount of cinnamic acid was detected by GC. Similar to the reaction of **1a** and acrylate **2a** (see Section 2.1), prolonged reaction time resulted in dicoupling product **8a** as a major product rather than **3** (entries 1–4). The maximum yield of **7a** was attained in 1.5 h (entries 1). The best yield of **7a** was obtained when the reaction was carried out by using the excess amount of **1a** (30 mmol) to **6a** (1.5 mmol). However, the yield of **7a** was still good to high when the amount of **1** was reduced to 20 mmol (entry 5). The addition of DBM as a ligand was effective in the present reaction and the yield of **7a** in the absence of a DBM ligand resulted in a decrease of the yield of **7a** to 33% (entry 6). As for a ligand, the use of benzoylacetone led to a similar result as that of DBM (entry 7). When acetylacetone (acacH) which is an effective ligand in the reaction of **1a** and **2a** (section 2.1) was employed instead of DBM, longer reaction time is needed to obtain the same catalytic activity (entries 8 and 9). The addition of the base is indispensible in the reaction and the reaction in the absence of Na_2_CO_3_ resulted in a considerable decrease of the yield of **7a** and **8a**, and several unidentified products (probably oligomers of **6a**) were formed (entry 10). A slight decrease of the yield of **7a** was observed when NaOAc was added as a base (entry 11). 

Furthermore, the effect of the HPMoV was examined under the reaction conditions as entry 1, Table 3 (entries 12–16). The best result was obtained by using H_4_PMo_10_VO_40_·26H_2_O to afford **7a** in 59% yield as shown in entry 1. Increase of the V content in the HPMoV was found to bring about a decrease of the yields of **7a** (entries 12–14). Moreover, vanadotungstphosphoric acid (H_5_PW_10_V_2_O_40_·27H_2_O) did not catalyze the present coupling reaction at all (entry 15). H_3_PMo_12_O_40_·30H_2_O not containing a vanadium atom was less efficient catalyst than HPMoV (entry 16).

In this reaction, acrolein (**6a**) was rapidly consumed when the reaction started, and the coupling product **7a** was reached maximum after 1.5 h, and gradually decreased because of further coupling with **1a** leading to dicoupling product **8a**. To confirm this sequential pathway for the formation of **8a**, an independent reaction of **7a** with **1** under these conditions gave **8a** in 61% yield. 

Several substituted benzenes were reacted with **2** under varying conditions and the results are summarized in Table 4.

The reaction of toluene (**1b**) with **6a** afforded a mixture of structural isomers **7b** (*o*:*m*:*p* = 13:44:43) in 59% yield along with small amounts of dicoupling products (entry 1). In the reaction with *t*-butylbenzene (**1h**) having bulky alkyl group, *m*- and *p*-products were obtained and *o*-product was not detected at all (entry 2). Anisole (**1c**) was subjected to react with **6a** to give a mixture of the corresponding coupling products **7d** (*o*:*m*:*p* = 17:10:73) in 45% total yield (entry 3). The reaction of 1,2-dimethoxybenzene (**1f**) with **6a** proceeded in high regioselectivity to produce 3,4-dimethoxycinnamaldehyde (**7e**) (45%), and the yield of the isomer, 2,3-dimethoxycinnamaldehyde, was found to be less than 2% (entry 4). In contrast, the reaction of 1,2-methylenedioxybenzene (**1i**) with **6a** led to a 45:55 mixture of 2,3- and 3,4-methylenedioxycinnnamaldehydes (**7f** and **7f’**) in 45% yield (entry 5). 

The oxidative coupling of methacrolein (**6b**) with **1a** was carried out under the conditions as entry 1, Table 3 (Equation 3).

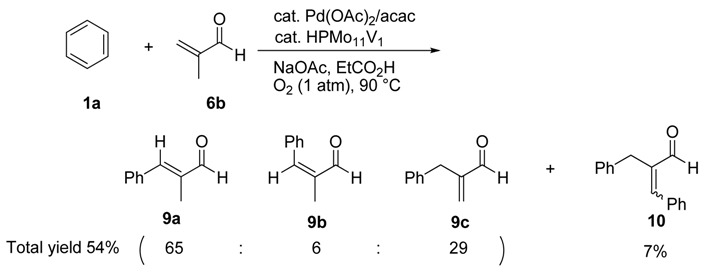
(3)

**Scheme 2:**
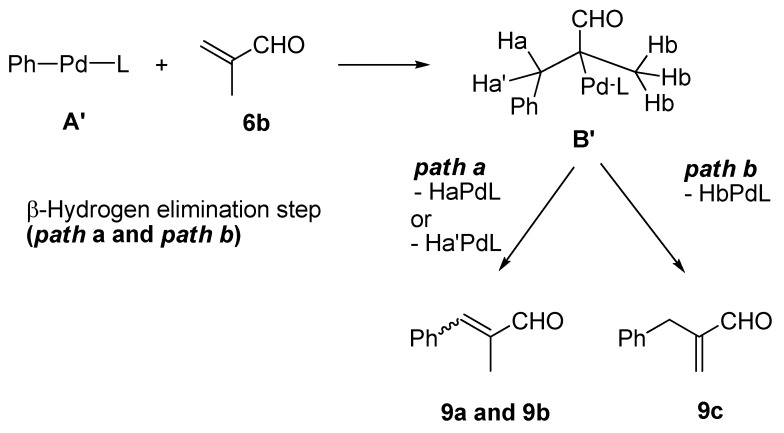
Formation of **9a**, **9b**, and **9c** from phenyl-palladium intermediate (**B’**).

In this reaction, a mixture of three isomeric coupling products consisting of **9a**, **9b** and **9c** were obtained in 54% yield (**9a**:**9b**:**9c** = 65:6:29) along with the dicoupling product **10** (7%). Based on the reaction mechanism as illustrated in Scheme 1, the reaction would proceed through a phenyl-palladium intermediate (**B’**) formed by the insertion of phenyl-palladium σ-complex (**A’**) to **6b**. Therefore, **9a, 9b** and **9c** can be formed by the following β-Hydrogen elimination step via path **a** (**9a** and **9b**: elimination of Ha or Ha’) and path **b** (**9c**: elimination of Hb) (Scheme 2) [12].

### 2.3. Oxidative Mizoroki-Heck type coupling of benzenes with ethylene

Synthesis of styrene by the oxidative arylation of ethylene with benzene under mild reaction conditions is also an attractive in organic synthesis, because the styrene is produced by the Friedel-Crafts reaction of benzene with ethylene followed by dehydrogenation of the resulting ethylbenzene. We performed the aerobic oxidative coupling reaction of benzene (**1a**) with ethylene (**11**) catalysed by the Pd(OAc)_2_/H_4_PMo_11_VO_40_·15H_2_O system, and styrene (**12**) was obtained as a major adduct. The results under various reaction conditions are summarized in Table 5.

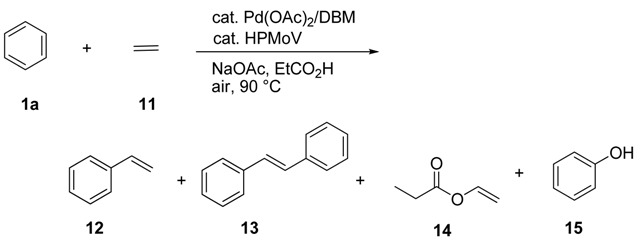
(4)

The reaction of benzene (**1a**) (30 mmol) was performed with a mixed gas of ethylene (**11**) (0.9 atm, *ca.* 2.0 mmol) and air (1.6 atm) in the presence of Pd(OAc)_2_ (10 μmol, *ca.* 0.5 mol % based on **11**), H_4_PMo_11_VO_40_·15H_2_O (10.3 mg, *ca.* 5 μmol), NaOAc (25 μmol), dibenzoylmethane (DBM) (30 μmol) and propionic acid (2 mL) at 90 °C for 8 h. As a result, styrene (**12**) was obtained in 202 μmol corresponding to 20 turnover numbers of Pd (TON = 20) with concomitant formation of *trans*-stilbene (**13**) in 25 μmol (TON = 5.2), and vinyl propionate (**14**) in 50 μmol (TON = 5.0), and phenol (**15**) in 30 μmol (TON = 3.0) (entry 1). Similar to the result reported in the reaction of benzene with acrylates and acrolein, the addition of ligand such as DBM brought about the improvement of the catalytic activity. The amount of DBM was reduced from 30 μmol to 10 μmol, the yield of **12** was decreased to 75 μmol (TON = 7.5) (entry 3). The reaction in the absence of DBM gave **12** in low yield and the catalyst was deactivated by forming the Pd black during the reaction course. As a ligand, acetylacetone (acacH) showed the similar catalytic activity for the formation of **12** (entry 4). The addition of the base is necessary to achieve the reaction and the reaction was sluggish in the absence of NaOAc (entry 5). The use of other base such as LiOAc or KOAc instead of NaOAc resulted in the lower TON of Pd for the formation of **12** (entries 6 and 7). Alternatively, Na_2_CO_3_ can also be used as an efficient base in the present reaction (entry 8).

The reaction was markedly influenced by the concentrations of ethylene and air, and the coupling reaction of **1a** with **11** was conducted under several varying ethylene/air pressures (entriy 1 *vs.* entries 9–12). Under the reaction of ethylene/air = 0.9 atm/1.6 atm, TON of Pd for **12** attained maximum (TON = 20) (entry 1). Thus, when the reaction of was performed under lower pressure of ethylene (ethylene/air = 0.5 atm/1 atm), the yield of **12** was decreased to 142 μmol (TON = 14) (entry 9). Furthermore, the amount of vinyl propionate **14** was increased with increasing of ethylene pressure. The yields of **14** was 190 μmol (TON = 19) and 243 μmol (TON = 24) when the reaction was carried out under ethylene/air = 3.6 atm/6.6 atm and 5.4/9.6 atm, respectively (entries 10–11). The reaction under high pressure of air (29.1 atm) with 0.9 atm of ethylene resulted in slightly lower catalytic activity (TON = 14) (entry 12).

As mentioned in the previous section, the role of HPMoV is important as the reoxidation system of the reduced Pd(0) catalyst Therefore, the reaction was performed by the use of HPAs under these conditions. In this reaction, HPAs having various V contents such as H_5_PMo_10_V_2_O_40_·28H_2_O, H_6_PMo_9_V_3_O_40_·30H_2_O and H_7_PMo_8_V_4_O_40_·28H_2_O can be used as reoxidation catalysts and afforded **12** in comparable yields with that of H_4_PMo_11_VO_40_·15H_2_O (entries 13 to 15). It was found that H_3_PMo_12_O_40_·30H_2_O not including V ion was found to serve a good reoxidation catalyst (entries 16–18) and the highest total TON of Pd for the formation of **12** and **13** was reached to 167, under the optimum reaction conditions (entry 18). In addition, various heteropoly acids such as H_4_PMo_11_WO_40_·27H_2_O, H_3_PMo_10_W_2_O_40_·29H_2_O, and H_4_SiMo_12_O_40_·27H_2_O showed similar catalytic activities (entries 19–21). However, the heteropoly acid without molybdenum ion such as H_5_PW_10_V_2_O_40_·27H_2_O was found to be inactive for the present reaction (entry 22). It is noteworthy that phenol (**15**) was not produced in the reaction using heteropoly acids not including V ion. By judging of these results, it is considered that molybdenum ion in the heteropoly acids is an essential component to promote the reoxidation of the reduced Pd(0) to Pd(II) for the present reaction. 

## 3. Experimental 

### 3.1. General procedure for oxidative coupling of benzene *(**1a**)* with ethyl acrylate *(**2a**)* (entry 1, Table 1)

A solution of Pd(OAc)_2_ (0.1 mmol), H_4_PMo_11_V_1_O_40_·30H_2_O (HPMo_11_V_1_) (46.7 mg, *ca*. 0.02 mmol), NaOAc (0.08 mmol), acetylacetone (0.1 mmol), benzene (**1a**) (30 mmol) and ethyl acrylate (**2a**) (1.5 mmol) in propionic acid (5 mL) was placed in a round bottom flask (30 mL) equipped with a balloon filled with O_2_, and the mixture was allowed to react under stirring at 90 °C for 2.5 h. The reaction gave ethyl cinnamate (**3a**), *β*-phenylcinnamate (**4a**) and 3-propionylacrylate (**5**) in 74%, 14% and 5% yields, respectively. All yields were determined by GLC analysis using nonane as internal standard. The products were characterized by ^1^H- and ^13^C-NMR and GC-MS, respectively.

### 3.2. General procedure for oxidative coupling of benzene *(**1**)* with ethylene *(**11**)* (entry 1 in Table 5)

**Caution**: In order to avoid an explosion of a mixture of ethylene and air, the reaction was carried out at the ethylene concentration deviated from explosion limits ranging in 3.1 to 32%. To a 50 mL stainless steel autoclave equipped with a magnetic stir bar were placed Pd(OAc)_2_ (10 μmol), H_4_PMo_11_VO_40_·15H_2_O (10.4 mg, *ca*. 5 μmol), NaOAc (25 μmol), dibenzoylmethane (DBM) (30 μmol), and benzene (**1a**) (30 mmol) and propionic acid (2 mL). To the autoclave was introduced with 0.9 atm of ethylene gas (**11**) and 1.6 atm of air, and the mixture was stirred at 90 °C for 8 h. All yields were detected by GLC analysis using dodecane as internal standard. The products were identified through a comparison of these analytical data with those of authentic samples.

## 4. Conclusions

In conclusion, we have shown the first direct oxidative coupling of benzenes with acrylates, α,β-unsaturated aldehydes, and ethylene under atmospheric dioxygen and air by using Pd(OAc)_2_ combined with HPMoV. In this catalytic system, addition of ligand such as acetylacetone (acacH) and dibenzoylmethane (DBM) was effective to suppress the Pd(0) black precipitation. All the presented oxidative coupling reactions in this review uses molecular oxygen as terminal oxidant. Therefore, these reactions provide very useful organic transformations from environmental and economical points of view.

## Figures and Tables

**Table 1:** Oxidative coupling of benzene (**1a**) with ethyl acrylate (**2a**) catalyzed by Pd(II)/HPMoV. ^a^

Entry	HPMoV	Base	Yield/% ^b^		
			3a	4a	5
1	H_4_PMo_11_VO_40_·30H_2_O	NaOAc	74	13	4
2^c^	H_4_PMo_11_VO_40_·30H_2_O	NaOAc	6	81	nd^d^
3	H_4_PMo_11_VO_40_·30H_2_O	None	6	nd^d^	4
4 ^e^	H_4_PMo_11_VO_40_·30H_2_O	NaOAc	53	2	8
5 ^e,f^	H_4_PMo_11_VO_40_·30H_2_O	NaOAc	75	16	1
6^g^	H_4_PMo_11_VO_40_·30H_2_O	NaOAc	51	5	16
7^h^	H_4_PMo_11_VO_40_·30H_2_O	NaOAc	20	nd ^d^	nd ^d^
8	None	NaOAc	9	nd ^d^	1
9	H_5_PMo_1__0_V_2_O_40_·28H_2_O	NaOAc	73	14	5
10	H_6_PMo_9_V_3_O_40_·30H_2_O	NaOAc	70	13	5
11	H_7_PMo_8_V_4_O_40_·28H_2_O	NaOAc	62	4	5
12	H_3_PMo_12_O_40_·30H_2_O	NaOAc	43	1	4
13	H_4_PMo_11_VO_40_·30H_2_O	LiOAc	66	5	7
14	H_4_PMo_11_VO_40_·30H_2_O	CsOAc	13	nd ^d^	1
15	H_4_PMo_11_VO_40_·30H_2_O	KOAc	11	nd ^d^	nd ^d^
16	H_4_PMo_11_VO_40_·30H_2_O	NH_4_OAc	12	nd ^d^	nd ^d^
17 ^i^	H_4_PMo_11_VO_40_·30H_2_O	NaOAc	74	8	6
18 ^j^	H_4_PMo_11_VO_40_·30H_2_O	NaOAc	10	Nd ^d^	3

^a^ A mixture of **1a** (30 mmol) and **2a** (1.5 mmol) was reacted in the presence of Pd-catalyst (0.1 mmol, 6.7 mol %), HPMoV (0.02 mmol, 1.3 mol %), base (0.08 mmol, 5.3 mol %) and acetylacetone (0.1 mmol, 6.7 mol %) in EtCOOH (5 mL) at 90 °C for 2.5 h. ^b^ GC yield based on **2a** used. ^c^ Reaction time was 5 h. ^d^ Not detected by GC. ^e^ The reaction was performed in the absence of acetylacetone. ^f^ Pd(acac)_2_ was used as catalyst. ^g^ **1a** (6 mmol) was used. ^h^ AcOH was used instead of EtCOOH. ^i^ The reaction was performed under air (1 atm) for 5 h. ^j^ The reaction was performed under argon (1 atm).

**Table 2:** Oxidative coupling of various arenes **1** and acrylates **2** catalyzed by Pd(II)/HPMoV. ^a^

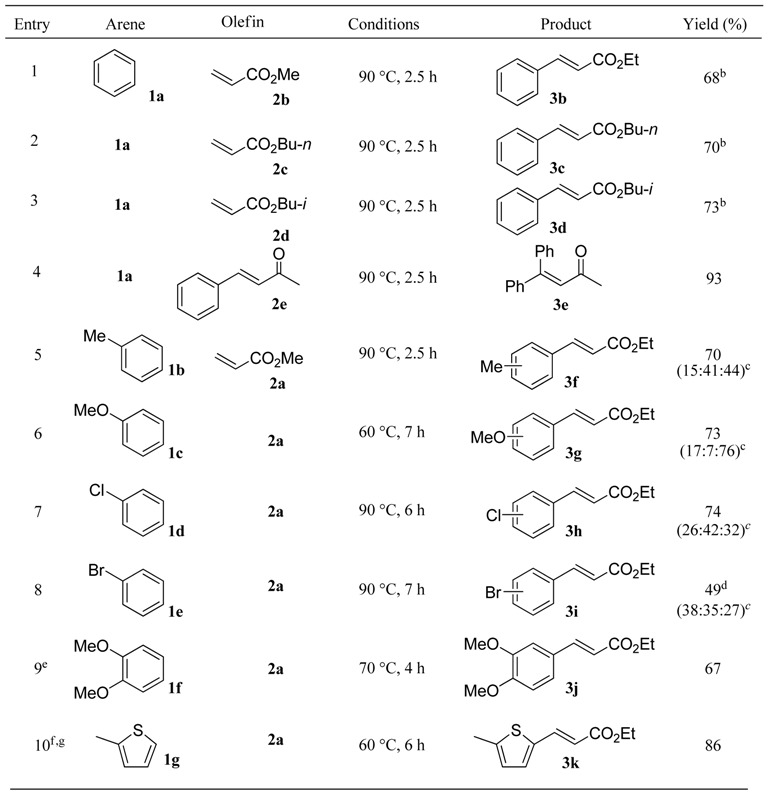

^a^ A mixture of arene **1** (30 mmol) and olefins **2** (1.5 mmol) was reacted in the presence of Pd-catalyst (0.1 mmol, 6.7 mol %), H_4_PMo_11_VO_40_·30H_2_O (0.02 mmol, 1.3 mol %), NaOAc (0.08 mmol, 5.3 mol %) and acetylacetone (0.1 mmol, 6.7 mol %) in EtCOOH (5 mL). ^b^ β-methylcinnamates are formed in 11–16% yields as by-products. ^c^ Ratio of *o:m:p*. ^d^ Ethyl cinnamate was obtained in 5%. ^e^ Arene (5 mmol) was used. ^g^ Arene (3 mmol) was used. ^h^ 5 wt % Pd(OAc)_2_/C was used instead of Pd(OAc)_2_.

**Table 3:** Oxidative coupling of benzene (**1a**) with acrolein (**6a**) catalyzed by Pd(II)/HPMoV. ^a^

Entry	HPMoV	Ligand	Time/h	Yield/% ^b^	
				7a	8a
1	H_4_PMo_11_VO_40_·26H_2_O	DBM	1.5	59	5
2	H_4_PMo_11_VO_40_·26H_2_O	DBM	1	26	nd ^d^
3	H_4_PMo_11_VO_40_·26H_2_O	DBM	2	45	18
4	H_4_PMo_11_VO_40_·26H_2_O	DBM	3	17	40
5^c^	H_4_PMo_11_VO_40_·26H_2_O	DBM	1.5	53	6
6	H_4_PMo_11_VO_40_·26H_2_O	None	1.5	33	nd ^d^
7	H_4_PMo_11_VO_40_·26H_2_O	benzoylacetone	1.5	52	6
8	H_4_PMo_11_VO_40_·26H_2_O	acacH	1.5	48	1
9	H_4_PMo_11_VO_40_·26H_2_O	acacH	2	54	8
10^e^	H_4_PMo_11_VO_40_·26H_2_O	acacH	2	3	nd ^d^
11^f^	H_4_PMo_11_VO_40_·26H_2_O	acacH	2	44	2
12	H_5_PMo_1__0_V_2_O_40_·28H_2_O	DBM	1.5	54	6
13	H_6_PMo_1__0_V_3_O_40_·30H_2_O	DBM	1.5	47	6
14	H_7_PMo_8_V_4_O_40_·28H_2_O	DBM	1.5	35	1
15	H_5_PW_1__0_V_2_O_40_·27H_2_O	DBM	1.5	nd^d^	nd ^d^
16	H_3_PMo_1__2_O_40_·30H_2_O	DBM	1.5	24	nd ^d^

^a^ A mixture of **1a** (30 mmol) and **6a** (1.5 mmol) was reacted in the presence of Pd(OAc)_2_ (0.1 mmol, 6.7 mol %), HPMoV (0.02 mmol, 1.3 mol %), Na_2_CO_3_ (0.05 mmol, 3 mol %) and ligand (0.1 mmol, 6.7 mol %) under O_2_ (1 atm) in EtCOOH (5 mL). ^b^ GC yield based on **6a** used. ^c^ **1a** (20 mmol) was used. ^d^ Not detected by GC. ^e^ The reaction was performed in the absence of Na_2_CO_3_. ^f^ NaOAc (0.08 mmol) was used instead of Na_2_CO_3_.

**Table 4:** Oxidative coupling of arenes **1** with acrolein (**6a**) catalyzed by Pd(II)/HPMoV. ^a^

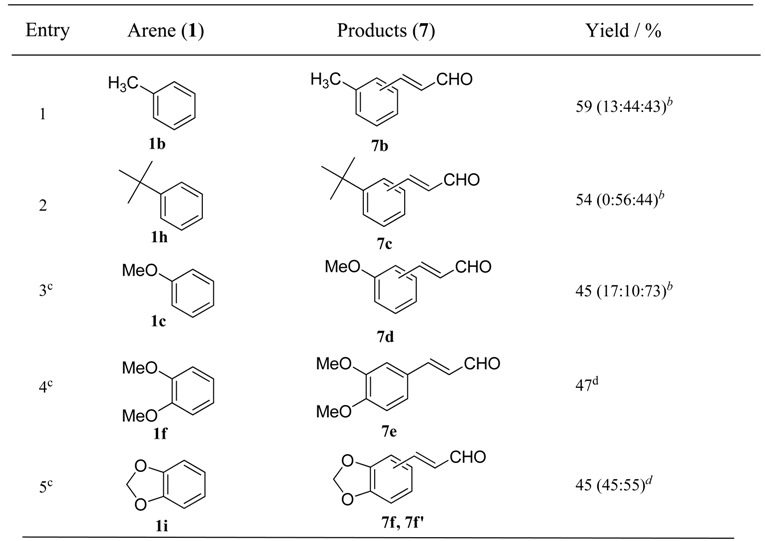

*^a^* A mixture of **1** (30 mmol) and **6a** (1.5 mmol) was reacted in the presence of Pd(OAc)_2_ (0.1 mmol, 6.7 mol %), H_4_PMo_11_VO_40_·26H_2_O (0.02 mmol, 1.3 mol %), Na_2_CO_3_ (0.05 mmol, 3 mol %) and DBM (0.1 mmol, 6.7 mol %) under O_2_ (1 atm) in EtCOOH (5 mL) at 90 °C, 1.5 h. ^b^ Ratio of *o*-:*m*-:*p*-isomer. ^c^ **1** (10 mmol) was reacted with **6a** (1.5 mmol) by Pd(OAc)_2_ (0.1 mmol, 6.7 mol %), H_4_PMo_11_VO_40_·26H_2_O (0.02 mmol, 1.3 mol %), Na_2_CO_3_ (0.05 mmol, 3 mol %) and acacH (0.1 mmol, 6.7 mol %) under O_2_ (1 atm) in EtCOOH (5 mL) at 90 °C, 1 h. *^d^* 2,3-Dimethoxycinnamaldehyde was obtained 2% yield as by-product. *^e^* Ratio of 2,3-methylenedioxy cinnamaldehyde (**7f**) to 3,4-methylenedioxy cinnamaldehyde (**7f’**).

**Table 5:** Oxidative coupling of benzene (**1a**) with ethylene (**11**) catalyzed by Pd(II)/HPMoV. ^a^

entry	HPMoV	Base	TON^b^ (μmol)			
			12	13	14	15
1	H_4_PMo_11_VO_40_·15H_2_O	NaOAc	20 (202)	5.2 (25)	5.0 (50)	3.0 (30)
2^c^	H_4_PMo_11_VO_40_·15H_2_O	NaOAc	4.6 (46)	nd^d^	8.3 (82)	4.5 (45)
3^e^	H_4_PMo_11_VO_40_·15H_2_O	NaOAc	7.5(75)	0.9 (4)	6.0 (59)	3.9 (39)
4^f^	H_4_PMo_11_VO_40_·15H_2_O	NaOAc	14 (138)	4.8 (24)	3.8 (38)	4.9 (49)
5	H_4_PMo_11_VO_40_·15H_2_O	None	1.6 (16)	nd^d^	2.0 (20)	nd^d^
6	H_4_PMo_11_VO_40_·15H_2_O	LiOAc	9.1 (90)	2.6 (13)	2.9 (29)	2.9 (29)
7	H_4_PMo_11_VO_40_·15H_2_O	KOAc	5.9 (59)	0.8 (4)	1.7 (17)	1.4 (14)
8^g^	H_4_PMo_11_VO_40_·15H_2_O	Na_2_CO_3_	19 (188)	9.7 (48)	5.2 (52)	7.1 (71)
9^h^	H_4_PMo_11_VO_40_·15H_2_O	NaOAc	14 (142)	4.0 (20)	3.4 (33)	-^i^
10^j^	H_4_PMo_11_VO_40_·15H_2_O	NaOAc	12 (119)	nd^d^	19 (190)	-^i^
11^k^	H_4_PMo_11_VO_40_·15H_2_O	NaOAc	5.3 (53)	nd^d^	24 (243)	-^i^
12^l^	H_4_PMo_11_VO_40_·15H_2_O	NaOAc	14 (137)	4.9 (24)	5.2 (52)	-^i^
13	H_5_PMo_1__0_V_2_O_40_·28H_2_O	NaOAc	16 (154)	4.2 (21)	4.4 (44)	3.8 (38)
14	H_6_PMo_9_V_3_O_40_·30H_2_O	NaOAc	17 (166)	7.7 (38)	2.9 (29)	4.0 (40)
15	H_7_PMo_8_V_4_O_40_·28H_2_O	NaOAc	19 (192)	4.9 (2.4)	6.4 (64)	3.6 (36)
16	H_3_PMo_1__2_O_40_·30H_2_O	NaOAc	22 (223)	11 (53)	2.9 (29)	0.3 (3)
17^m^	H_3_PMo_1__2_O_40_·30H_2_O	NaOAc	42 (420)	4.4 (22)	3.5 (35)	nd^d^
18^m,n^	H_3_PMo_1__2_O_40_·30H_2_O	NaOAc	100 (1003)	67 (335)	3.9 (39)	nd^d^
19	H_3_PMo_1__1_WO_40_·27H_2_O	NaOAc	24 (239)	12 860)	3.0 (30)	nd^d^
20	H_3_PMo_1__0_W_2_O_40_·29H_2_O	NaOAc	23 (228)	7.8 (39)	2.8 (28)	nd^d^
21	H_4_SiMo_1__2_O_40_·27H_2_O	NaOAc	22 (218)	7.8 (39)	2.9 (29)	nd^d^
22	H_5_PW_1__0_V_2_O_40_·27H_2_O	NaOAc	1.5 (15)	trace	nd^d^	nd^d^

^a^ A mixture of benzene (**1a**), Pd(OAc)_2_ (10 μmol), H_4_PMo_11_VO_40_·15H_2_O (10.3 mg, *ca.* 5 μmol), NaOAc (25 μmol), dibenzoylmethane (DBM) (30 μmol) was allowed to react under 0.9 atm of etyhylene (**11**) and 1.6 atm of air in EtCOOH (2 mL) at 90 °C for 8 h. ^b^ Turnover number (TON) based on Pd(OAc)_2_ used. ^c^ The reaction was performed in the absence of dbm. ^d^ Not detected by GC. ^e^ Dbm (10 μmol) was used. ^f^ Acetylacetone was used instead of dbm. ^g^ Na_2_CO_3_ (13 μmol) was used. ^h^ The reaction was performed under ethylene/air = 0.5 atm/1 atm. ^i^ Not determined. ^j^ The reaction was performed under ethylene/air = 3.6 atm/6.4 atm. ^k^ The reaction was performed under ethylene/air = 5.4 tm/9.6 atm. ^l^ The reaction was performed under ethylene/air = 0.9 atm/29.1 atm. ^m^ **1a** (120 μmol) was reacted with 0.9 atm of **11** in the presence of Pd(OAc)_2_ (10 μmol), HPMo_11_V_1_ (6.8 μmol), NaOAc (32 μmol), and dbm (120 μmol) in EtCOOH (8 mL) under 1.6 atm of air using a 120 mL autoclave. ^n^ The reaction was performed at 120 °C.
